# Comparison of Primers for the Detection of *Phytophthora* (and Other Oomycetes) from Environmental Samples

**DOI:** 10.3390/jof8090980

**Published:** 2022-09-19

**Authors:** Treena I. Burgess, Diane White, Sarah J. Sapsford

**Affiliations:** 1 Phytophthora Science and Management, Harry Butler Institute, Murdoch 6150, Australia; 2 School of Biological Science, University of Canterbury, Christchurch 8401, New Zealand

**Keywords:** environmental DNA, phylogeny, DNA barcoding, high-throughput nucleotide sequencing, plant pathogen

## Abstract

Many oomycetes are important plant pathogens that cause devastating diseases in agricultural fields, orchards, urban areas, and natural ecosystems. Limitations and difficulties associated with isolating these pathogens have led to a strong uptake of DNA metabarcoding and mass parallel sequencing. At least 21 primer combinations have been designed to amplify oomycetes, or more specifically, *Phytophthora* species, from environmental samples. We used the Illumina sequencing platform to compare 13 primer combinations on mock communities and environmental samples. The primer combinations tested varied significantly in their ability to amplify *Phytophthora* species in a mock community and from environmental samples; this was due to either low sensitivity (unable to detect species present in low concentrations) or a lack of specificity (an inability to amplify some species even if they were present in high concentrations). Primers designed for oomycetes underestimated the *Phytophthora* community compared to *Phytophthora*-specific primers. We recommend using technical replicates, primer combinations, internal controls, and a phylogenetic approach for assigning a species identity to OTUs or ASVs. Particular care must be taken if sampling substrates where hybrid species could be expected. Overall, the choice of primers should depend upon the hypothesis being tested.

## 1. Introduction

Many oomycetes are significant plant pathogens in agricultural fields, orchards, urban environments, and natural ecosystems [1,2]. Some of the most important pathogens of natural ecosystems belong to the genus *Phytophthora* [3]. The impacts post-introduction of these pathogens to naïve ecosystems have resulted in irreversible changes to plant species composition [4]. Many historical introductions accompanied the global expansion of agriculture with colonisation, while more recent introductions follow the plants-for-planting trade [5,6,7]. Endemic oomycetes are likely to have a more benign role within coevolved vegetation and play a role in shaping plant distribution [8].

Isolation of oomycetes as pathogens from diseased material is relatively straightforward, but isolation from soil is a complicated process [9]. Recovering oomycetes from rhizosphere soil (or other plant material) involves covering the material with water and floating bait leaves on top; motile zoospores swim to the surface and infect the bait, which are then plated onto selective media. *Phytophthora* is commonly isolated using this method, and antimicrobials may be included in the media to suppress the growth of *Pythium* [10]. A sample may include several *Phytophthora* species, but only the dominant species are usually isolated [11]. Sarker et al. [11] demonstrated competitive exclusion, whereby the species that rapidly produce sporangia were most frequently isolated. Due to low recoveries and lack of reproducibility, this methodology is unsuitable for studying community ecology and dynamics [11].

Alternatively, DNA metabarcoding and mass parallel sequencing has emerged as a technology suitable for such studies [12,13,14], and has been embraced by oomycete researchers (Table 1) with varying success. Early studies using generic ITS primers detected a limited number of oomycetes [15], while studies using the *Phytophthora*-specific primers developed by [16] have detected numerous species in a range of environments [17,18]. These studies have enriched knowledge on oomycete biology and ecology, providing baseline data [19] or a more nuanced analysis of environmental filtering [20].

The first metabarcoding papers on *Phytophthora* published in 2013 used the 454 pyrosequencing platform [15,21]. Recent publications have used Illumina or Pac Bio platforms. Based on a literature search, 21 different primer combinations have been used in various studies, all purported to be *Phytophthora* or oomycete-specific (Table 1). Legeay et al. [22] compared three primer sets and found genus-specific primers developed by Scibetta et al. [16] to be a reliable tool for the qualitative description of environmental *Phytophthora* communities. However, a more comprehensive comparison testing for reproducibility, specificity, and sensitivity is required of the different primer sets using a single sequencing platform.

In the current study, we compared 13 of the 21 primer combinations using the Illumina sequencing platform. The study was conducted in four separate Illumina runs over three years, expanding to compare newly published primer sets. We compared several primers in each run but always included those of Scibetta et al. [16], as adapted for metabarcoding by Català et al. [23]. We tested mock communities created from known DNA concentrations across the *Phytophthora* phylogeny and previously characterised environmental samples. Amplification was conducted in triplicate, and the replicates were given individual barcodes to determine the importance of replication in the amplification step of the process. The purpose of this study was to answer the following questions: (1) Are the primers specific to oomycetes and/or *Phytophthora*? (2) Are the reads obtained related to the amount of DNA? (3) Do the primers successfully amplify environmental samples? (4) Are technical replicates necessary?

## 2. Materials and Methods

### 2.1. Mock Communities and Environmental Samples

Isolates were grown on ½ PDA, and DNA was extracted from mycelia using Quick DNA™ Fungal/Bacterial MiniPrep kit. DNA concentration was determined using Qbit kits (Invitrogen Qubit™ dsDNA HS Assay Kit). Mock *Phytophthora* communities were created using 1 μL of DNA from each species regardless of the DNA concentration. There was over a 200 X difference between the highest and the lowest concentrations. Species included were from across the *Phytophthora* phylogeny [24]. Three mock communities were generated: the first, containing DNA of 50 species, was used for the first two metabarcoding runs; the second community, with 66 species, was used in the third run; and the third mock community, with 61 species, was used in the fourth run (Figure S1). Additionally, environmental DNA (eDNA) samples previously determined to contain various *Phytophthora* species were also amplified alone (runs 1–4) and together with the mock communities (runs 2–4). Individual eDNA samples from the study of Khdair et al. [25] collected from parks in Perth, Western Australia were combined to generate four eDNA mixes (E1–4) expected to contain 16, 16, 26, and 32 species, respectively.

**Table 1:** A summary of metabarcoding studies in chronological order conducted to determine diversity or community dynamics of oomycetes.

Publication	Gene Region ^1^	Primer Set ^2^	Study Location and Scale	Number of Samples	Sequencing Platform	% Oom	% Phyt	Species Detected
Coince et al. [15]	O-ITS	P14	**France:** Beech forest	20 root samples 20 soil samples	454	0.8%		2 *Pythium* 2 *Phytophthora*
Vannini et al. [21]	O-ITS	P3	**Italy:** Chestnut forests in the Latium region	10 soil samples	454	78%		15 *Phytophthora* 18 oomycetes
Català et al. [23]	P-ITS	P4	**Spain:** Forests and plantations in northern Spain	24 soil samples 15 water samples	454		>99%	35
Sapkota and Nicolaisen [26]	O-ITS	P14	**Denmark:** Agricultural field and carrots showing symptoms	26 soil samples 11 carrot samples	454	95%		2 *Phytophthora* 65 oomycete
Agler et al. [27]	O-ITS	P15	**Germany:** Phyllosphere of wild *Arabidopsis thaliana* populations	5 sites, two harvests	Illumina	na		Genus only
Prigigallo et al. [28]	P-ITS	P4	**Italy:** Soil and root samples from 8 potted nurseries	8 pooled samples	454		>99%	25 *Phytophthora*
Riit et al. [29]	O-ITS	P1 ^3^	**Estonia:** Plant nurseries and surrounds	20 soil samples	Illumina	25%		Genus only
Burgess et al. [19] Burgess et al. [30]	P-ITS	P4	**Australia:** 5 states, soil samples from natural ecosystems	640 soil samples	454		>99%	68
Català et al. [17]	P-ITS	P4	**Spain:** Two oak forests in eastern Spain	23 soil samples 10 root samples	454		>99%	13 *Phytophthora*
Cerri et al. [31]	O-ITS	P14	**Italy:** 5 freshwater ecosystems, some with reed dieback	96 root, rhizosphere and soil samples	454	88%		523 OTUs ^4^
Bose et al. [32]	P-ITS	P4	**South Africa:** Four sites from *Eucalyptus* and *Acacia* plantations and adjacent forests; soil samples	120 soil samples	454		>99%	32 *Phytophthora*
Redondo et al. [20]	P-ITS	P16	**Sweden:** 96 sites in 16 rivers over 2 years	192 water samples (filtered)	PacBio	74%		36 *Phytophthora*
Gómez et al. [33]	O-ITS	P3	**Spain:** Declining oak	52 soil samples	Illumina	50%		178 ASVs ^5^
Legeay et al. [22]	P-ITS	P11	**Mock Community:** 24 species of *Phytophthora* and other fungi, eukaryotes, and bacteria	Mock communities	Illumina		>99%	19 *Phytophthora*
Legeay et al. [22]	P-ITS	P11	**France:** Rhizosphere soil	8 eDNA samples	Illumina		95%	7 *Phytophthora*
Legeay et al. [22]	O-ITS	P17	**Mock Community:** 24 species of *Phytophthora* and other fungi, eukaryotes, and bacteria	Mock communities	Illumina	100%		21 *Phytophthora*
Legeay et al. [22]	O-ITS	P17	**France:** Rhizosphere soil	8 eDNA samples	Illumina	97%		1 *Phytophthora*
Legeay et al. [22]	O-RAS ^6^	P18	**Mock Community:** 24 species of *Phytophthora* and other fungi, eukaryotes, and bacteria	Mock communities	Illumina		100%	22 *Phytophthora*
Mora-Sala et al. [34]	P-ITS	P4	**Spain:** 6 *Quercus ilex* stands in 3 regions	150 soil samples 365 bait leaves	454		>99%	37 *Phytophthora*
Redekar et al. [35]	O-ITS	P3	**USA:** Recycled irrigation water in a nursery across 12 months	302 water filters	Illumina	6%		48 *Phytophthora* >50 oomycetes
Redekar et al. [35]	O-ITS	P3	**USA:** Recycled irrigation water in a nursery across 12 months	82 bait leaves	Illumina	55%		26 *Phytophthora* 21 oomycetes
Riddell et al. [18]	P-ITS	P4	**Britain:** 14 gardens/amenity woodland sites	140 soil samples	Illumina		>99%	35 *Phytophthora*
Sapp et al. [36]	O-*cox*2	P13	**Spain:** Andalusia, 22 trees in declining oak stands	66 root samples	Illumina	n/a ^7^		n/a ^7^
Foster et al. [37]	O-ITS	P14	**USA:** Microbiome of roots of three cultivars of Rhododendron grown under different conditions in four nurseries	120 root balls	Illumina	n/a		3 *Phytophthora* 4 *Pythium*
Green et al. [38]	P-ITS	P4	**Britain:** 14 gardens/amenity woodland sites	140 soil samples	Illumina		>99%	23 *Phytophthora*
Khdair et al. [25]	P-ITS	P4	**Australia:** Parks and gardens in one city	236 soil samples	454		>99%	44 *Phytophthora*
Legeay et al. [39]	P-ITS	P11	**French Guiana:** Two sites in rainforest; 10 plots and up to 10 host families at each plot	93 soil samples 264 bait leaves	Illumina		>99%	6 *Phytophthora*
Maciá-Vicente et al. [40]	O-*cox*2	P19	**Germany:** Naturally co-occurring Brassicaceae	146 soil and root samples	Illumina	n/a		951 ASVs
Noel et al. [41]	O-ITS	P3	**USA:** Soyabean rhizosphere communities (roots) 4 genotypes, 4 plots, and 6 replicates	362 rhizosphere samples	Illumina	20% of ASVs		86% *Pythium* 3% *Phytophthora*
Redekar et al. [42]	O-ITS	P3	**USA:** Recycled irrigation water in a nursery across 12 months	168 water ilters and leaf baits	Illumina	50%		32 *Phytophthora* >50 oomycetes
Riddell et al. [43]	P-ITS	P4	**Britain:***Phytophthora* in water samples in juniper woodland (rain traps and rivers) over 12 months	36 pooled water samples (filtered)	Illumina		>99%	14 *Phytophthora*
Bose et al. [44]	P-ITS	P4	**South Africa:** Four sites from *Eucalyptus* and *Acacia* plantations and adjacent forests, root samples	120 root samples	454		>99%	27 *Phytophthora*
Fiore-Donno and Bonkowski [45]	O-ITS	P20	**Germany:** 3 established biodiversity sites; 50 grassland and 50 forest at each	300 soil samples	Illumina	96%		31% known species
Gyeltshen et al. [46]	P-ITS	P4	**Australia:** Topsoil stockpiles (3) and adjacent forest	42 bulk root samples from 20 plants species	Illumina		>99%	23 *Phytophthora*
Khaliq et al. [47]	P-ITS	P4	**Australia:** Altitude survey, 3 roads, 20 sites per road, sample at disturbed edge and 50 m into natural vegetation	120 pooled root samples	Illumina		>99%	25 *Phytophthora*
Landa et al. [48]	P-ITS	P4	**Britian:** 14 sites—9 disturbed, 5 undisturbed	132 soil samples	Illumina		100%	62 *Phytophthora*
Landa et al. [48]	O-*cox1*	P5	**Britian:** 14 sites—9 disturbed, 5 undisturbed	132 soil samples	Illumina	71%	11%	52 *Phytophthora*
Marčiulynienė et al. [49]	O-ITS	P14 ^8^	**Lithuania:** 5 different tree species in 7 bare root forest nurseries	350 root samples 350 soil samples	PacBio	1.5%		2 *Phytophthora* 33 oomycete
Rossmann et al. [50]	O-ITS	P15	**Norway:** Soil from internationally shipped plants	73 soil samples (before and after enrichment)	Illumina	72%	5%	Genus only
Green et al. [51]	P-ITS	P4	**Britain:** Water and root samples from nurseries	400 water and root samples	Illumina	na	na	63 *Phytophthora*

^1^ O = oomycete-specific, P = *Phytophthora*-specific; ^2^ see Table 2; ^3^ used the correct ITS1oo primer [52], but reported sequence in published manuscript as that ascribed to P1 in Table 2; ^4^ no attempt was made to assign OTUs to species level; ^5^ only some ASVs were identified to species level; ^6^ primers failed to amplify eDNA; ^7^ Materials and Methods state ‘after subtraction of non-oomycete taxa’, but no percentage supplied, ^8^ PCR1 only with ITS6 and ITS4.

### 2.2. Comparison of Primer Combination

Thirteen primer combinations (primer sets) were compared across four Illumina metabarcoding runs (Table 2). All runs included the well-defined *Phytophthora*-specific primer set (P4) [16] used previously by our laboratory [19,30,32,65]. Primers included those that amplify the internal transcribed spacer (ITS), cytochrome oxidase subunit 1 (*cox*I), cytochrome oxidase subunit 2 (*cox*2), and the 40S ribosomal protein S10 (*rps*10) gene regions.

No-template negative PCR controls were included each time a PCR reaction was set up, and for nested PCR protocols, these were carried forward to the second round in the same manner as for the samples. If a band was visualised in these negative PCR controls, the products were discarded. The first-round PCR was conducted in triplicate (technical replicates), and replicates were assigned a unique barcode. In the first metabarcoding run, four primer combinations (P1–4) were compared using DNA from mock community MIX1 and two eDNA samples (E1 and E2), resulting in 36 amplicons, each uniquely barcoded. In the second run, two primer combinations (P4–5) were compared using DNA from mock community MIX1, two separate eDNA samples (E1 and E2), and one eDNA sample (E1) spiked with mock community MIX1, resulting in 24 amplicons, each uniquely barcoded. In the third run, six primer combinations were compared (P4, P6–10; P8 and P9 failed to amplify) using DNA from mock community MIX2, one eDNA sample (E3) and the same eDNA sample spiked with mock community MIX2, resulting in 36 amplicons, each uniquely barcoded. In the fourth run, four primer combinations were compared (P4, P11–13; P12 failed to amplify) using DNA from the mock community MIX3, one eDNA sample (E4), and the same eDNA sample spiked with mock community MIX3, resulting in 27 amplicons, each uniquely barcoded. All mixes were made in a ratio of eDNA to MIX of 20:1.

PCR conditions differed between primer sets. The PCRs for primer sets P1, P2, P3, P7, P9, and P12 were performed in 25 μL-volume tubes containing 12.5 μL of PCR buffer KAPA HiFi HotStart ReadyMix (KAPA Biosystems, Wilmington, MA, USA), 8 μL of PCR grade water, 1 μM of each primer, and 2.5 ul μL of genomic DNA. PCR cycling conditions were 94 °C for 2 min, 30 cycles of 95 °C for 20 s, 60 °C for 25 s, and 72 °C for 1 min, before a final 72 °C for 7 min and holding at 4 °C. The PCRs for primer set 13 were performed in 25 μL-volume tubes containing 12.5 μL of PCR buffer KAPA HiFi HotStart ReadyMix (KAPA Biosystems), 6.5 μL of PCR grade water, 1 μM of each primer,1 ul of Bovine Serum Album10 mg/mL (Fisher Biotech, Perth, Australia), and 3 μL of genomic DNA. PCR cycling conditions were 94 °C for 4 min, 36 cycles of 95 °C for 40 s, 55 °C for 40 s, and 72 °C for 1 min, before a final 72 °C for 5 min and holding at 4 °C. The PCRs for primer sets P5, P6, P8, and P10 were performed in 25 μL-volume tubes containing 12.5 μL of PCR buffer KAPA HiFi HotStart ReadyMix (KAPA Biosystems), 7.5 μL of PCR grade water, 1 μM of each primer and 2.5 μL of genomic DNA (first round) or 2.5 μL of the PCR1 product. The mix was the same for P4 and P11, except that 32.5 μL of genomic DNA was used in the first round. PCR cycling conditions for P4, P6, P8, and P11 were 94 °C for 2 min, 30 cycles of 95 °C for 20 s, 60 °C for 25 s, and 72 °C for 1 min, before a final 72 °C for 7 min, and holding at 4 °C. PCR cycling conditions for P5 were 95 °C for 5 min, 35 cycles of 94 °C for 40 s, 52 °C for 40 s, and 72 °C for 1 min, before a final 72 °C for 10 min and holding at 4 °C. PCR cycling conditions for P10 for PCR1 were 94 °C for 2 min, 35 cycles of 94 °C for 30 s, 59 °C for 45 s, and 72 °C for 1 min, before a final 72 °C for 10 min and holding at 4 °C; and for PCR2, they were 94 °C for 2 min, 30 cycles of 95 °C for 20 s, 60 °C for 25 s, and 72 °C for 1 min, before a final 72 °C for 7 min and holding at 4 °C

Amplicon library preparation was performed according to recommended protocols (Illumina Demonstrated Protocol: 16S Metagenomic Sequencing Library Preparation) with some exceptions. PCR products were visualised on 1% agarose gels and pooled based on DNA concentrations as quantified using Qbit kits. Uniquely indexed libraries were pooled for the sequencing run, which was performed on Illumina MiSeq using 500-cycle V2 chemistry (250 bp paired-end reads) following the manufacturer’s recommendations.

### 2.3. Bioinformatic Analysis

Paired-end reads were merged using USEARCH v10 [66] with a minimum overlap length of 50 bp with no gaps allowed in the merged alignments. Only forward reads were used for Primer sets P1 and P5. Sequence deconvolution, such as quality control and clustering, was also carried out using USEARCH v10. Specifically, sequences less than 200 bp and of low mean quality (<20) were removed. Sequences that passed quality control were clustered into operational taxonomic units (OTUs) with a similarity threshold of 99%.

Blast searches were conducted in Geneious Prime^®^ 2019.2.3 (https://www.geneious.com), and OTUs identified during the bioinformatic analysis were divided into two folders; oomycetes and non-oomycetes. No further analysis was conducted on the non-oomycete reads. Oomycetes were divided into *Phytophthora* and other oomycetes. Where possible, species identity was assigned to all *Phytophthora* OTUs using phylogenetic analysis and a curated *Phytophthora* database in Geneious. This was performed by assigning an OTU to one of the 12 phylogenetic clades recognised within the genus and then creating sequence alignments, including the sequence of the type isolates of all described species (as designated by Abad et al. [24]) using the MAFFT algorithm in Geneious. Also included in these alignments were the sequences of isolated but as-yet-undescribed species from Australia and sequences recognised as putative new species in other Australian metabarcoding studies [19]. A simple phylogenetic analysis was conducted using Geneious tree builder. Species identity was assigned to an OTU if the sequence identity was >99 identical and fell into a strongly supported terminal clade with a known taxon. Other oomycetes were only identified to the genus level, except for *Phytopythium litorale*, which had been included in the mock communities. The hybrid *P.* × *alni* was included in the mock community and could be distinguished in the ITS1 gene region based on the amplification of both parental alleles.

### 2.4. Statistical Analysis

All analyses were conducted using R 4.1.0 (https://www.R-project.org/). To determine how sensitive and quantitative each primer set was, we compared the number of reads of each species found in the species mix ‘mock’ communities to the DNA concentration of each species using a negative binomial generalised linear model with function glm.nb. Our response was the number of reads, and our predictors were DNA concentration (log-transformed) and primer set. Each run was analysed separately. Function ‘emmeans’ was used to run post hoc pairwise comparisons of primer sets. Model assumptions were verified by visually inspecting residuals for assumptions of normality and homoscedasticity [67].

We used the Bray–Curtis dissimilarity index to calculate dissimilar values from OTU relative abundance data to determine differences in the community composition of oomycete communities from different primer sets on environmental samples and among replicates. Bray-Curtis dissimilarity values were calculated with the vegdist function in package vegan [68]. Communities were visualised using the nonmetric multidimensional scaling (NMDS) function ‘metaMDS’. To test for community differences across the primers, environmental samples, and replicates, permutational multivariate analysis of variance (PERMANOVA) using the function adonis was used in package vegan [68]. Run (i.e., 1–4) was a factor used as a ‘strata’ to control for different run and sequencing conditions. The null hypothesis of a PERMANOVA is that the centroids of the groups are equivalent for all groups. We repeated the above steps using the Jaccard dissimilarity index to calculate dissimilarly values from OTU presence–absence data.

To compare how well primers worked on environmental samples, we also compared alpha diversity (i.e., species richness). Each run was analysed separately. We conducted linear models using function ‘lm’ where alpha diversity was the response and primer set was the predictor. Model assumptions were verified by visually inspecting residuals for assumptions of normality and homoscedasticity [67].

To determine whether spiking of environmental samples with species mix ‘mock’ communities improved sequence results, we conducted nonparametric paired t-tests using function wilcox.test (i.e., Wilcoxon signed-rank test) between the percentage of reads of environmental communities and the percentage of reads of environmental sampled spiked mock communities. A nonparametric test was used as the assumption of normality failed with our response (i.e., percentage of reads).

## 3. Results

### 3.1. Phytophthora Detection in Mock Communities

In run 1, DNA concentration (*z-value* = 16.34, *p* < 0.001) was a significant indicator of sequence number. However, each primer differed in the amount of sequences generated from the mock community (*p* < 0.001). P4 performed the best and P1 performed the worst as a function of DNA concentration (Figure S2A). In run 2, DNA concentration (*z-value* = 7.809, *p* < 0.001) was a significant indicator of sequence number. However, P5 significantly differed to P4 in the amount of sequences generated from the mock community (P5 *z-value* = −3.989, *p* < 0.001). P4 performed better than P5 as a function of DNA concentration (Figure S2B). In run 3, DNA concentration (*z-value* = 7.352, *p* < 0.001) was a good indicator of sequence number. Primers differed in the amount of sequences generated from the mock community (*p* < 0.001); specifically, Primers P4 and P6 (*z-ratio* = 3.698, *p* = 0.012) and Primers P6 and P7 differed significantly (*z-ratio* = −3.949, *p* = 0.0005) between each other (Figure S2C). In Run 4, DNA concentration (*z-value* = 3.414, *p* < 0.001) was a good indicator of sequence number. However, primers preformed similarly and did not differ between each other (*p* = 0.652) (Figure S2D).

**Figure 1:**
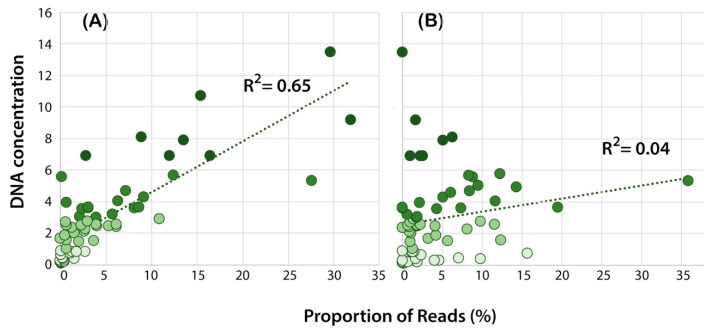
Relationship between the DNA concentration (ng/μL) and the proportion of reads for each *Phytophthora* species in the mock community (MIX2) for (**A**) amplification with ITS primer set P4 and (**B**) amplification with rps10 primer set P10. The data points are coded based on the DNA concentration as per Figure 2; the darker colours correspond to higher DNA concentration. The R^2^ value is for the goodness of fit based on simple linear regression.

Overall, for the mock communities, there was a positive correlation between DNA concentration and the number of reads in the sample for many of the primer sets based upon the ITS gene region (P1–P4, P8) (Table 3). The correlation was weak for ITS primer sets P6 and P11, the *cox* primers sets P5 and P13, and the *rps*10 primer set P10 (Table 3, Figure 1).

The primer sets differed in their ability to amplify species within the mock communities. Primer set P4 could amplify 77–94% of species across the four runs (Table 3). ITS primers sets P2, P3, and P7 produced similar results, successfully amplifying most of the species in the mock community (Table 3). ITS primer set P1 amplified 76% of species, ITS primer set P11 amplified 56% of the species, *cox*1 primer set P5 amplified 50% of species, *cox*2 primer set P13 amplified 77% of the species, and *rps*10 primer set P10 amplified 84% of species (Table 3).

**Figure 2:**
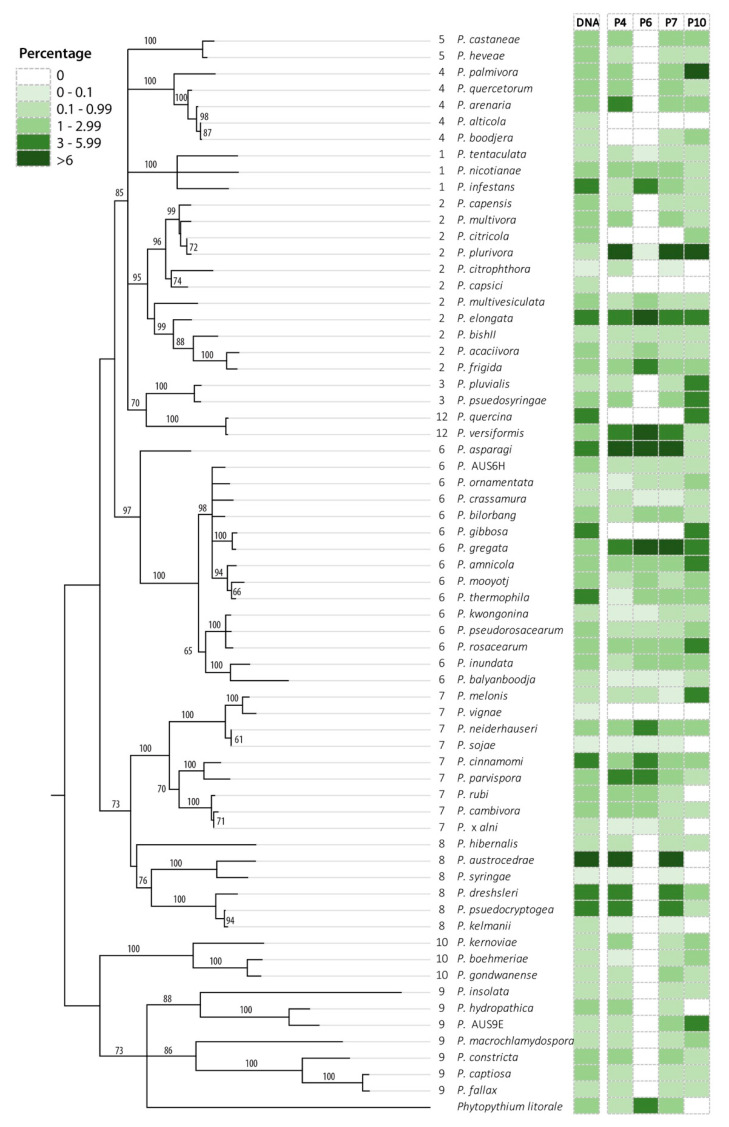
Phylogenetic representation based on ITS gene region of the *Phytophthora* species in the mock community MIX2. The relative proportion of DNA of each species (as a percentage) and the average relative abundance of reads obtained for each species (as a percentage) are colour-coded as per the legend.

For the primer sets based upon the ITS gene region (P1–P4, P6, P8, P11), the inability to detect some cryptic species is because they have an identical sequence for the short fragment of the gene region amplified (Figure 2, Figures S3–S6). These species were (a) *P. alticola* and *P. boodjera*, (b) *P. citricola* and *P. plurivora*, (c) *P. gregata* and *P. gibbosa*, and (d) *P. versiformis* and *P. quercina*. Based on phylogeny, it should be possible to distinguish these species using the primer sets for other gene regions (P5, P10, and P13), and indeed both *P. gregata* and *P. gibbosa* were detected using all these primer pairs, *P. versiformis* and *P. quercina* were also detected using primer sets P10 and P13 (Figure 2, Figures S3 and S5), and *P. citricola* and *P. plurivora* were detected by primer set P10 (Figure S4). However, P5 and P10 could not pick up several other species from the mock community, so the lack of detection of the closely related species may not be based on the specificity of the primers but on primer sensitivity. The hybrid *P.* × *alni* could be recognised by primer sets based on the ITS gene region as both ITS alleles amplified and closely matched *P. uniformis*, a species not included in the mock community. However, while they could be correctly assigned to *P.* × *alni* in a mock community, this would not be possible for an environmental sample as the ITS1 sequence of the *P.* × *alni* alleles cannot be separated from that of *P. uniformis.*

### 3.2. Phytophthora Detection in eDNA Samples Spiked with the Mock Community

eDNA samples were spiked with the mock community in runs 2–4 (Figures S4–S6). As expected, when eDNA samples were spiked with the mock community, the primer sets that were specific to oomycetes when used with eDNA samples alone were specific when these samples were spiked; these were primer sets P4, P6, and P10. For primer set P7, only 2.4% of reads were from oomycetes; for the *cox*1 primer, this was 83%; while for the *cox*2 primer set P13, it was 98.5%.

Mock community MIX1 and eDNA sample E1 share 13 *Phytophthora* species, so the total number of species possible to detect is 53 (Figure S4). Primer set P4 detected 46 species but missed 6 species from the mock community and 1 from the eDNA sample (Table 3, Figure S4). Primer set P5 detected only 25 species; 25 were missed from the mock community and 5 from the eDNA sample (Table 3, Figure S4). Mock community MIX2 and eDNA sample E3 share 25 *Phytophthora* species, so the total number possible to detect is 67 (Figure S4). Primer set P4 detected 57 species but missed 10 species from the mock community and 2 from the eDNA sample (Table 3, Figure S5). Primer set P5 detected only 30 species; 37 were missed from the mock community and 10 from the eDNA sample (Table 3, Figure S5). Primer set P7 (even though only 2.4% of reads were oomycete) detected 41 *Phytophthora* species, but missed 26 species from the mock community and 3 from the eDNA sample (Table 3, Figure S5). Primer set P10 detected 51 species, but missed 16 species from the mock community and two from the eDNA sample (Table 3, Figure S4). Mock community MIX3 and eDNA sample E4 share 30 species, so the total number possible to detect is 64 species (Table 3, Figure S6). Primer set P4 detected 38 species, but missed 22 from the mock community and 9 from the eDNA sample. Primer set P11 detected 36 species but missed 24 from the mock community and 10 from the eDNA sample. Primer set P13 detected 48 species, but missed 12 from the mock community and 7 from the eDNA sample.

In most cases, the *Phytophthora* species not detected were present at the lower concentrations (<1% of the *Phytophthora* DNA in the mock community) (Figures S3–S6). The exception for this is the P5 primer set (cox1 gene region) which failed to amplify several species present in a higher concentration (Figure S4), P6 primer set, which did not amplify numerous species from clades 4, 5, 8, 9 and 10 (Figure S5) and primer set P11, which gave poor amplification of species in clades 6–8 (Figure S6).

### 3.3. Phytophthora Detection in eDNA Samples

For eDNA samples, the primer sets differed considerably in the percentage of reads that could be attributed to *Phytophthora* (and/or oomycetes). Primer sets P4, P6, P10, and 11 are highly specific and only amplify oomycetes (>99.9% of reads), with primer set P4 primarily targeting only *Phytophthora* (Table 3). Primer set P5 does target oomycetes (40% for sample E1 and 60% for sample E2) but also amplified algal and some plant DNA, while primer sets P1, P2, P3, and P7 are not specific, with oomycetes accounting for less than 1% of the total read number in the environmental samples. For primer set P13, 3.25% of the reads were oomycete. For both these *cox* primer pairs, P5 and P13, fewer of the reads were from oomycetes when amplifying the eDNA sample alone than when spiked, so it appears that while being specific, they are less sensitive when faced with low levels of oomycete DNA in an eDNA sample.

Of the 16 *Phytophthora* species previously detected from sample E1, 11 were detected by primer set P4 in run 1 and 15 in run 2; the other primer sets only detected between 2–4 species each (Table 3, Figures S3 and S4). Of the 16 Phytophthora species previously detected in sample E2, 13 were detected by primer set P4 in run 1 and 11 in run 2; primer set P2 detected 7 species, primer set P3 detected 3 species, P5 detected 3 species, while primer set P1 failed to detect any *Phytophthora* species in the sample (Table 3, Figures S3 and S4). Of the 26 *Phytophthora* species previously detected from sample E3 (used for run 3), 23 were detected by primer set P4, 16 by primer set P6, 11 by primer set P7, and 10 by primer set P10 (Table 3, Figure S5). Of the 33 *Phytophthora* species previously detected from sample E3, 29 were detected by primer set P4, 23 by primer set P11, and 13 by primer set P13 (Table 3, Figure S6).

**Figure 3:**
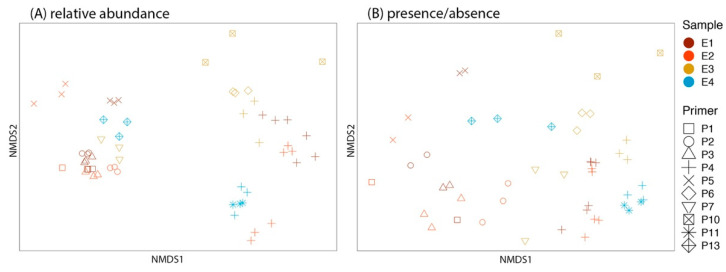
The nonmetric multidimensional scaling (NMDS) plot demonstrates the community composition of environmental samples (i.e., E1–E4) amplified by 10 primer sets. Each sample was run in triplicate. Community composition from (**A**) relative abundance data (stress = 0.174) and (**B**) presence–absence data (stress = 0.173).

Community composition from the relative abundance data differed depending on the primer used (F_9,56_ = 7.889, R^2^ = 0.56, *p* = 0.0001) and among the different environmental samples (F_3,56_ = 3.748, R^2^ = 0.09, *p* = 0.001). Replicates did not differ from each other (F_2,56_ = 0.839, R^2^ = 0.01, *p* = 0.558) (Figure 3A). Community composition from the presence absence data differed depending on the primer used (F_9,56_ = 8.290, R^2^ = 0.59, *p* = 0.0001) and among the different environmental samples (F_3,56_ = 4.672, R^2^ = 0.11, *p* = 0.001). The interaction between primer and sample was also significant (F_4,56_ = 4.145, R^2^ = 0.13, *p* = 0.001). Replicates did not differ from each other (F_2,56_ = 0.452, R^2^ = 0.007, *p* = 0.896) (Figure 3B).

**Figure 4:**
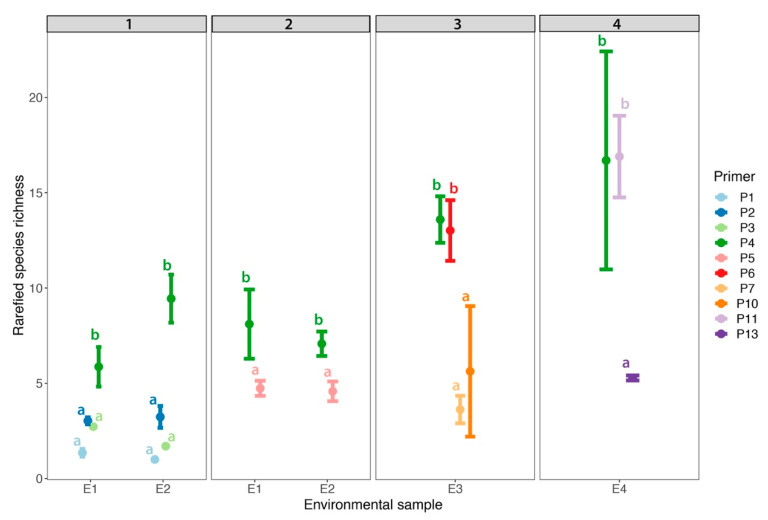
Mean rarefied species richness of environmental samples (i.e., E1–E4) across three replicates for 10 different primers. Each panel demonstrates the primers used across 4 sequencing runs. Letters indicate significant differences among primers in each run (i.e., each run was analysed separately). Points represent the means ± standard deviations.

In addition to community composition, we also compared alpha diversity (i.e., species richness) among the primers. In run 1, P4 resulted in the highest alpha diversity (*t-value* = 8.705, *p* < 0.001) compared to all other primer sets (Figure 4). Alpha diversity of P1, P2, and P3 (*p* > 0.05) did not differ among each other. In run 2, P5 resulted in lower alpha diversity than P4 (*t-value* = −4.983, *p* < 0.001; Figure 4). In run 3, P4 and P6 resulted in similar estimates of alpha diversity (t-ratio = 0.378, *p* = 0.98) and P7 and P10 resulted in similar estimates of alpha diversity (t-ratio = 1.246, *p* = 0.618). Overall, P4 and P6 resulted in higher alpha diversities than P7 and P10 (Figure 4). In run 4, P4 and P11 resulted in similar estimates of alpha diversity (*t-value* = −0.055, *p* = 0.958); however, P13 produced much lower estimates in comparison to P4 and P11 (*t-value* = −4.00, *p* < 0.001; Figure 4).

### 3.4. Technical Replicates

For the mock communities, technical replicates generally amplified the same species. Additionally, there was no significant difference between technical replicates for the environmental samples (Figure 3). However, while the differences between replicates were not significant, there were differences between samples, particularly in the relative proportion of reads assigned to each species. For example, Table 4 presents the relative proportion of reads for each *Phytophthora* species detected across the three replicates for eDNA sample E3. Individual replicates all failed to detect some species; this differed so that when combined, a greater number of species were detected (Table 4). Overall, while the dominant species were found in all replicates, the percent of total reads varied considerably (Table 4).

## 4. Discussion

### 4.1. Comparison of Primers

The primer pairs tested, which had all previously been used to study oomycete communities, varied greatly in their ability to amplify *Phytophthora* species in a mock community and from environmental samples. Each of the four Illumina sequencing runs included a mock community. We deliberately used a range of DNA concentrations (200 × difference between highest and lowest concentration) to test the limits of detection of the primers and determine if there was any relationship between DNA concentration and the number of reads. Using nested PCR on these mock communities, DNA concentration was a good indicator of read number for the *Phytophthora*-specific primers of Scibetta et al. [16]; for the remaining primers, the relationship was poor. This was due to either low sensitivity (an inability to detect species present in low concentrations) or a lack of specificity (an inability to amplify some species even if they were present in high concentrations). The number of rDNA copies varied widely among fungi, ranging from about 14 to 1442 copies [69]. A similar variation could be expected in ITS copy numbers between *Phytophthora* species, and this would impact the relative quantification of the species.

Overall, the ITS primers detected more species than those based on other gene regions. In general, most of the primers tested amplified most species in the mock community, but some primers also failed to amplify whole clades from within the *Phytophthora* phylogeny, in particular the ITS primers P6 [70] and the *cox*1 primers [48]. When there was a background of other organisms (eDNA spiked with a mock community), most of the primers amplified the same *Phytophthora* species as they had for the mock community alone, with a slight decrease in sensitivity.

Several studies have also included mock communities, although never with as many species. Català et al. [23] mixed similar DNA concentrations of eight species and found that the reads obtained for one species, *P. plurivora*, were much lower than expected. Legeay et al. [22] generated a mock community of 25 species, including two hybrids, with and without a background community of other microorganisms, and tested these with three primers. Amplification was different between the primer sets; *P. plurivora* was preferentially amplified, and other species were completely missing depending on the primer set used. The mock community of Sapkota and Nicolaisen [26] included seven species with the DNA mixed in different ratios; two species failed to amplify, and the number of reads correlated poorly with DNA concentration. Riddell et al. [18] used two mock communities—one with 15 species, the other with 10 species—to compare two sequence analysis tools (Bowtie and Swarm). Swarm correctly detected more species from the mock community, but Bowtie produced fewer false positives.

The most successful assays used a nested approach to amplify environmental DNA. The primers designed by Scibetta et al. [16] are *Phytophthora*-specific. Of the purported oomycete-specific primer pairs, the ITS primers (P6) used for direct sequencing of eDNA samples (not metabarcoding) by Dickie et al. [70] and the *rps*10 primers of Martin et al. [71] only amplified oomycetes in environmental samples. The other ITS primers tested (P2, P3, and P7) are not specific, and <1% of the reads could be assigned to oomycetes. For the *cox*1 primers [48] and *cox*2 primers [36], 40% and 3% of the reads in environmental samples, respectively, could be assigned to oomycetes.

Foster et al. [58] designed oomycete-specific primers for *rps*10 gene regions and tested these on a mock community containing 24 oomycete species and diverse environmental samples in a single PCR round. These primers detected 23 species in the mock community, and oomycetes accounted for 99% of the reads amplified from the environmental samples. We were unsuccessful in amplifying environmental samples using a single PCR, and thus used a nested approach with the PRV primers of Martin et al. [59] in the first round and the new oomycete specific *rps10* primers of Foster et al. [58] in the second round. In our study, using the nested approach, the *rps10* primers amplified 83% of the *Phytophthora* species in the mock community. In their recent publication, the authors of [58] amended the PRV primers to sequence the oomycete database. If we had used the amended PRV primers in the first round, we might have amplified more species from the mock community.

The *Phytophthora*-specific primers designed by Scibetta et al. [16] are used in a nested PCR for environmental samples. Most studies use the species-specific primers first (Table 1); however, Legeay et al. [22] and Legeay et al. [39] use the specific primers second. When compared directly, the amplification across the *Phytophthora* phylogeny was superior when the specific primers were used first (P4).

Landa et al. [48] compared the *Phytophthora-*specific primers designed by Scibetta et al. [16], P4, and primers for *cox*1 gene region, P5, on 132 environmental DNA samples from disturbed sites in the UK. For the ITS primers, 93% of the reads were *Phytophthora*, and 20 species and 21 unknown phylotypes were detected; while for the *cox*1 primers, 71% of the reads were assigned to oomycetes, 16% to *Phytophthora*, and 12 species and 17 unknown *Phytophthora* phylotypes were detected by Landa et al. [48]. The results were in agreement only from two locations. Similarly, in the current study, using the *cox*1 primers, 40% of reads were assigned to oomycetes and 7.5% to *Phytophthora*. However, in two environmental samples, the *cox*1 amplification only detected 25% of the *Phytophthora* species found using the P4 ITS primers.

### 4.2. Technical Replicates

A small amount of target DNA within a sample (as is the case for *Phytophthora* DNA within environmental samples) can lead to PCR stochasticity during metabarcoding [72]. PCR replication (technical replicates) is seen as the way to maximise diversity detection as it offsets replicate variability and maximises diversity detection. In a very detailed study, Alberdi et al. [14] reported considerable diversity differences between PCR replicates from each environmental sample resulting from PCR stochasticity and/or accumulation of PCR and sequencing errors. They compared different approaches for combining the data: in the additive approach, the sequences from the three PCR replicates were added together; in the restrictive approach, only sequences present in two of the three replicates were included. The additive approach increases the likelihood of detecting rare taxa, while the restrictive approach reduces the chance of incorporating artificial sequences and results in lower diversity. We have used the additive approach in the current study.

### 4.3. Hybrid Species

While all primers have their strengths and weaknesses, none of them can likely provide a perfect mirror of the true oomyete community. Good experimental design, laboratory practices, and bioinformatics workflow all increase the reliability of results; however, there is one type of organism that cannot be detected by metabarcoding; hybrid species. This is particularly important for *Phytophthora* and other oomycetes, especially if water sampling is involved. As demonstrated in the current study, the hybrid *P. alni* could be assigned in the mock community because one of the alleles produced closely matched *P. uniformis*, one of the hybrid’s known parents [73,74]. However, these taxa could not be separated in an environmental sample where both *P. × alni* and *P. uniformis* could be present. This inability to detect hybrids will be the case for all environmental samples and is particularly important for water samples. Clade 6 *Phytophthora* species have an aquatic lifestyle and are abundantly recovered from water [35,75,76]. They readily hybridise, and hybrids characterised to date contain the mitochondrial DNA of one parent at the nuclear DNA of two [77,78,79]. The hybrids are often stable and undergoing concerted evolution [78]. Amplifying a multicopy gene such as ITS results in the alleles of the two parent species and mixed alleles with signatures of both parents [78]. Thus, in a metabarcoding study based on ITS locus, the ASVs from a hybrid could be assigned to two known species and additional ‘unknown species’. Only a single parent species will be detected if a mitochondrial gene is amplified. We recommend that the results from metabarcoding of *Phytophthora* from water should be treated with caution. Similarly, while hybrids from other *Phytophthora* clades may not be dominant in a particular environment, there are common pathogens of agricultural fields and nurseries, for example, within the *P. cryptogea* complex in clade 8 [80] and also among species in clade 1 [81]. If metabarcoding is conducted with two loci (one nuclear and one mitochondrial) and the sample contains a known hybrid with the nuclear loci of one parent and the mitochondrial loci of another, and both parents are absent, then that hybrid could be detected in an environmental sample.

## 5. Conclusions

Here, based on the results and observation of the current study, we make several recommendations on treating samples once in the laboratory.

1. Primers designed for oomycetes do not have the same sensitivity toward *Phytophthora* as the *Phytophthora*-specific primers. Studies that use oomycete-specific primers to study *Phytophthora* communities have probably underestimated *Phytophthora* diversity. The selection of primers is a trade-off between detecting *Phytophthora* or detecting oomycetes and will depend upon the study’s intent.
2. Our results show that using multiple primer sets would reduce taxonomic biases and increase taxonomic coverage.
3. While taking technical replicates separately through the process and assigning unique barcodes may be helpful, this could be an expensive option. We recommend conducting the PCR steps in triplicate and then combining them before adding barcodes.
4. Use a phylogenetic approach to assign OTUs or ASVs to species rather than simple blast searches. By doing so, minor sequencing errors that do not influence phylogenetic placement will allow several OTUs to be assigned to the same species.
5. Internal controls were not included in the current study but would be a valuable addition to any protocol. Green et al. [38] included four samples containing a mix of synthetic ‘*Phytophthora*’ sequences of known base composition on the plate as a check for sequence contamination. These can be synthetic reference sequences included in the initial PCR reactions as control samples to determine any cross-contamination during the amplification stage.
6. Many *Phytophthora* species can hybridise, especially those commonly found in water. Care must be taken with metabarcoding studies as it is not possible to identify hybrids.

## Figures and Tables

**Table 2:** Details of the primers used in four separate metabarcoding runs. Primers sets were coded P1 to P21. Nested PCR was conducted for primer sets P4, P5, P6, P8, and P11. Primer sets P14–21 were not tested, but were included as they related to the studies presented in Table 1. Amplicon size including primers (size) and the annealing temperature (AT) of the PCR reaction are also provided for primers used in the current study. Primer set P4 was included in all the runs for comparison purposes.

Code		D	Primer	Sequence	Size	AT	Run1	Run2	Run3	Run4	Reference for Primer
P1 ^1^ (O)	PCR1	F	ITS1oo	#F-GGA AGG ATC ATT ACC ACA	900	60	Y				Riit et al. [29]
		R	ITS4ngs	#R-GTC CTS CGC TTA TTG ATA TGC							Tedersoo et al. [53]
P2 (P)	PCR1	F	ITS1oo	#F-GGA AGG ATC ATT ACC ACA	350	60	Y				Riit et al. [29]
		R	ITS7	#R-GAG CGT TCT TCA TCG ATG TGC							Cooke et al. [54]
P3 (O)	PCR1	F	ITS6	#F-GAA GGT GAA GTC GTA ACA AGG	500	60	Y				Cooke et al. [54]
		R	ITS7	#R-GAG CGT TCT TCA TCG ATG TGC							Cooke et al. [54]
P4 (P)	PRC1	F	18Ph2F	GGA TAG ACT GTT GCA ATT TTC AGT	400	60	Y	Y	Y	Y	Scibetta et al. [16]
		R	5.8S1R	GCA RRG ACT TTC GTC CCY RC							Scibetta et al. [16]
	PCR2	F	ITS6	#F-GAA GGT GAA GTC GTA ACA AGG	350	60					Cooke et al. [54]
		R	5.8S1R	#R-GCA RRG ACT TTC GTC CCY RC							Scibetta et al. [16]
P5 ^1^ (O)	PCR1	F	COX1levup-F	TCA WCW MGA TGG CTT TTT TCA AC	nd	52		Y			Choi et al. [55]
		R	COX1levlo-R	CYT CHG GRT GWC CRA AAA ACC AAA							Choi et al. [55]
	PCR2	F	Hvshort-F	#F-GNA TGA AYA AYA THA GYT TYT GG	500	52					Landa et al. [48]
		R	COX1levlo-R	#R-CYT CHG GRT GWC CRA AAA ACC AAA							Choi et al. [55]
P6 (O)	PCR1	F	ITS6	GAA GGT GAA GTC GTA ACA AGG	nd	60			Y		Cooke et al. [54]
		R	ITS2P	GCA GCG TTC TTC ATC GAT GT							Znajda et al. [56]
	PCR2	F	OOMUP18Sc	#F-TGC GGA AGG ATC ATT ACC ACA C	350	60					Lievens et al. [57]
		R	5.8S1R	#R-GCA RRG ACT TTC GTC CCY RC							Scibetta et al. [16]
P7 (O)	PCR1	F	OOMUP18Sc	TGC GGA AGG ATC ATT ACC ACA C	400	60			Y		Lievens et al. [57]
		R	ITS2P	GCA GCG TTC TTC ATC GAT GT							Znajda et al. [56]
P8 ^2^ (O)	PCR1	F	ITS6	GAA GGT GAA GTC GTA ACA AGG	na	60			Y		Cooke et al. [54]
		R	5.8S1R	GCA RRG ACT TTC GTC CCY RC							Scibetta et al. [16]
	PCR2	F	OOMUP18Sc	#F-TGC GGA AGG ATC ATT ACC ACA C	na	60					Lievens et al. [57]
		R	5.8S1R	#R-GCA RRG ACT TTC GTC CCY RC							Scibetta et al. [16]
P9 ^2^ (O)	PCR1	F	rps10-F	#F-GTT GGT TAG AGY ARA AGA CT	550	59			Y		Foster et al. [58]
		R	rps10-R	#R-ATR YYT AGA AAG AYT YGA ACT							Foster et al. [58]
P10 (O)	PCR1	F	PRV9-F	GTT GGT TAG AGT AAA AGA CT	na	59			Y		Martin et al. [59]
		R	PRV9-R	GTA TAC TCT AAC CAA CTG AGT							Martin et al. [59]
	PCR2	F	rps10-F	#F-GTT GGT TAG AGY ARA AGA CT	550	59					Foster et al. [58]
		R	rps10-R	#R-ATR YYT AGA AAG AYT YGA ACT							Foster et al. [58]
P11 (P)	PCR1	F	Oom18S	GCG CAT CGT GCT AGG GAT AG	nd					Y	Legeay et al. [22]
		R	ITS7	GAG CGT TCT TCA TCG ATG TGC							Cooke et al. [54]
	PCR2	F	18Ph2F	#F-GAA GGT GAA GTC GTA ACA AGG	400						Scibetta et al. [16]
		R	5.8S1R	#R-GCA RRG ACT TTC GTC CCY RC							Scibetta et al. [16]
P12 ^3^ (O)	PCR1	F	ITS1oo(c)	#F-GGA AGG ATC ATT ACC ACAC						Y	Riit et al. [52]
		R	ITS7	#R-GAG CGT TCT TCA TCG ATG TGC							Cooke et al. [54]
P13 (O)	PCR1	F	Cox2hud-F	#F-GGC AAA TGG GTT TTC AAG ATC C						Y	Hudspeth et al. [60]
		R	Cox233D8r	#R-GAA TAT TCA TAR STC CAR TAC C							Sapp et al. [36]
P14 (O)	PCR1	F	ITS6	GAA GGT GAA GTC GTA ACA AGG							Cooke et al. [54]
		R	ITS4	TCC TCC GCT TAT TGA TAT GC							White et al. [61]
	PCR2	F	ITS6	#F-GAA GGT GAA GTC GTA ACA AGG							Cooke et al. [54]
		R	ITS7	#R-GAG CGT TCT TCA TCG ATG TGC							Cooke et al. [54]
P15 (O)	PCR1	F	ITS1O	#F-CGGAAGGATCATTACCAC							Thines et al. [62]
		R	5.8S-O-Rev	#R-AGCCTAGACATCCACTGCTG							Agler et al. [27]
P16 ^4^ (P)	PCR1	F	A2	ACT TTC CAC GTG AAC CGT TTC AA							Drenth et al. [63]
		R	I2	GAT ATC AGG TCC AAT TGA GAT GC							Drenth et al. [63]
P17 (O)	PCR1	F	DC6	GAG GGA CTT TTG GGT AAT CA							Cooke et al. [54]
		R	ITS7	GAG CGT TCT TCA TCG ATG TGC							Cooke et al. [54]
	PCR2	F	Oom18S	#F-GCG CAT CGT GCT AGG GAT AG							Legeay et al. [22]
		R	ITS7	#R-GAG CGT TCT TCA TCG ATG TGC							Cooke et al. [54]
P18 (O)	PCR1	F	Yph1F	#F-CGA CCA TKG GTG TGG ACT TT							Weir et al. [64]
		R	Yph2R	#R-ACG TTC TCM CAG GCG TAT CT							Weir et al. [64]
P19 (O)	PCR1	F	Cox2hud-F	#F-GGC AAA TGG GTT TTC AAG ATC C							Hudspeth et al. [60]
		R	Cox2-RC4	#R-TGA TTW AYN CCA CAA ATT TCR CTA CAT TG							Choi et al. [55]
P20 (O)	PCR1	F	S1777F	GGT GAA CCT GCG GAA GGA							Fiore-Donno and Bonkowski [45]
		R	5.8SOomR	TCT TCA TCG DTG TGC GAG C							Fiore-Donno and Bonkowski [45]
	PCR2	F	S1786StraF	#F-GCG GAA GGA TCA TTA CCA C							Fiore-Donno and Bonkowski [45]
		R	5.8SOomR	#R-TCT TCA TCG DTG TGC GAG C							Fiore-Donno and Bonkowski [45]
P21 (O)	PCR1	F	ITS3oo	#F-AGT ATG YYT GTA TCA GTG TC							Riit et al. [52]
		R	ITS4	#R-TCC TCC GCT TAT TGA TAT GC							White et al. [61]

#F = forward Illumina adaptor TCG TCG GCA GCG TCA GAT GTG TAT AAG AGA CAG; #R = reverse Illumina adaptor GTC TCG TGG GCT CGG AGA TGT GTA TAA GAG ACAG; ^1^ forward only analysed; ^2^ amplification failed for P8, amplification was only successful for the mock community for P9; ^3^ P12 was tested for run4, but after repeated attempts we could not produce an PCR product; ^4^ P16 not used in current study as amplicon is too long for Illumina.

**Table 3:** Summary data for four metabarcoding runs for the mock community (MIX1–3) and the four eDNA samples (E1-E4) and for the combination of MIX1+E1 included in run 2, MIX2+E3 included in run 3 and MIX3+E4 included in run 4.

Metabarcoding Run	Run1	Run1	Run1	Run1	Run2	Run2	Run3	Run3	Run3	Run3	Run4	Run4	Run4
**Primer Combination**	**P1**	**P2**	**P3**	**P4**	**P4**	**P5**	**P4**	**P6**	**P7**	**P10**	**P4**	**P11**	**P13**
**Gene Region Amplified**	**ITS**	**ITS**	**ITS**	**ITS**	**ITS**	**COX1**	**ITS**	**ITS**	**ITS**	**RPS**	**ITS**	**ITS**	**COX2**
**Sample**	**MIX1**	**MIX1**	**MIX1**	**MIX1**	**MIX1**	**MIX1**	**MIX2**	**MIX2**	**MIX2**	**MIX2**	**MIX3**	**MIX3**	**MIX3**
**Average number of reads**	6 684	11,196	23,109	37,755	14,511	4105	18,942	8756	24,607	10,045	13,003	13,594	13,179
***Phytophthora*** **species detected**	37	46	46	46	45	25	59	33	60	55	47	34	47
**Species missed from MIX**	13	4	4	4	5	25	7	33	6	11	14	25	14
**Relationship between reads and** **DNA concentration**	0.613	0.665	0.613	0.75	0.732	0.095	0.650	0.261	0.526	0.037	0.385	0.063	0.143
**Sample**	**E1**	**E1**	**E1**	**E1**	**E1**	**E1**	**E3**	**E3**	**E3**	**E3**	**E4**	**E4**	**E4**
**Average number of reads**	21,546	6002	28,349	12,084	27,587	6440	25,009	22,083	15,698	18,203	23,155	16,158	7879
**% *Phytophthora* reads**	0.02	0.14	0.06	100	99.93	7.49	100	99.72	0.17	41.55	99.73	87.16	3.25
**% Oomycete reads**	0.02	1.03	0.30	100	100	40	100	99.72	0.17	100	99.96	99.94	3.25
***Phytophthora*** **species detected**	2	2	3	11	15	4	23	16	11	10	29	23	13
**Species missed from eDNA sample**	14	14	15	5	1	11	3	10	15	16	3	9	19
**Sample**	**E2**	**E2**	**E2**	**E2**	**E2**	**E2**							
**Average number of reads**	11,326	8844	9142	65,551	19,638	3957							
**% *Phytophthora* reads**	0	0.15	0.04	77.83	100	44.21							
**% Oomycete reads**	0	0.16	0.05	100	100	60.37							
***Phytophthora*** **species detected**	0	7	2	13	11	3							
**Species missed from eDNA sample**	16	9	14	3	5	13							
**Sample**					**E1+** **MIX1**	**E1+** **MIX1**	**E3+** **MIX2**	**E3+** **MIX2**	**E3+** **MIX2**	**E3+** **MIX2**	**E4+** **MIX3**	**E4+** **MIX3**	**E4+** **MIX3**
**Average number of reads**					36,097	3247	21,696	11,490	10,001	16,514	16,136	14,872	15,740
**% *Phytophthora* reads**					100	76.44	99.87	97.56	2.41	88.05	99.61	99.41	99.48
**% Oomycete reads**					100	83	100	99.98	2.42	100	99.96	99.90	99.48
***Phytophthora*** **species detected**					46	25	57	30	41	51	38	36	48
**Species missed from MIX**					6	25	10	37	26	16	22	24	12
**Species missed from eDNA sample**					1	5	2	10	3	2	9	10	7

The mock community MIX1 is comprised of 49 species, MIX2 is comprised of 66 species, and MIX3 is comprised of 61 species; the environmental DNA samples E1 and E2 are comprised of 16 species, E3 is comprised of 26 species, and E4 is comprised of 32 species.

**Table 4:** Percentage of the total number of reads of each *Phytophthora* species detected for three technical replicates of environmental sample E3 amplified with primers P4, P6, and P10. The average percent of reads is also given (AV). Cells are colour-coded, as per Figure 2.

Phytophthora Species	Clade	P4	P4	P4	P4	P6	P6	P6	P6	P10	P10	P10	P10
		1	2	3	AV	1	2	3	AV	1	2	3	AV
P. nicotianae	1	20.5	10.4	7.32	12.3	7.52	10.2	0.01	3.03				
P. acaciivora	2	0.01	11.1	0.01	2.73	4.88	4.65	0.01	1.82				
P. capensis	2			1.61	0.70						0.89		0.25
P. elongata	2	10.9	0.04	0.02	3.49	0.03	0.06	0.01	0.02				
P. multivora	2	0.01	9.61	19.3	10.8	0.02			0.01				
P. plurivora	2	0.03	0.02	0.25	0.13						63.9	0.10	17.9
P. arenaria	4	5.92	12.9	8.70	8.84								
P. boodjera	4		1.39		0.34								
P. palmivora	4	4.45		10.5	5.99					0.05		29.9	13.1
P. amnicola	6	8.38	6.73	2.29	5.31	2.25	8.17	14.6	10.4				
P. asparagi	6	16.5	7.19	0.04	7.03	4.68	13.5	4.37	5.16				
P. bilorbang	6					0.02	6.91	0.07	0.57		2.05		0.57
P. gibbosa ^1^	6										23.0	0.03	6.44
P. gregata	6	0.15	0.09	0.01	0.07	7.02	0.20	4.22	4.76		0.12		0.03
P. inundata	6	0.01		0.02	0.01	0.02	0.04		0.01				
P. moyootj	6	0.01	0.02	0.01	0.01								
P. rosacearum	6		2.26	0.01	0.55			0.01	0.01				
P. thermophila	6					0.03	0.06	3.12	1.96			0.03	0.01
P. cambivora	7	0.03	5.18		1.28	4.65	7.67	6.64	6.12				
P. cinnamomi	7	9.02	0.04	0.01	2.88	12.1	6.70	0.01	4.16			6.85	2.98
P. niederhauserii	7	8.07	7.06	13.2	10.1	8.52	15.8	18.8	15.5			28.2	12.3
P. drechsleri	8	0.01	0.01	0.01	0.01						0.05	0.03	0.03
P. pseudocryptogea	8	0.01	0.02	7.02	3.08								
P. syringae	8	0.01	2.77	2.26	1.67								
P. AUS12A	12	15.8	23.1	27.5	22.7	44.3	12.7	42.6	40.9	99.9	9.87	34.8	46.4
P. versiformis	12	0.06		0.01	0.02	3.92	13.3	5.35	5.52				
No. species detected		20	19	21	23	15	14	14	16	2	7	8	11
No. species not detected		6	7	5	3	11	12	12	10	24	17	16	15

^1^ it is not possible to separate *P. gregata* and *P. gibbosa* with ITS primer sets P4 and P6.

## Data Availability

OTUs from each run will be made available from Mendelay data. doi:10.17632/b4nfr67932.1.

## Supplementary Materials

The following supporting information can be downloaded at: https://www.mdpi.com/article/10.3390/jof8090980/s1, Figure S1. Phylogeny based on the ITS gene region of the *Phytophthora* species was used to create the mock communities. Mock community MIX1 was used for metabarcoding runs 1 and 2, mock community MIX2 was used in metabarcoding run 3, and mock community MIX3 was used in metabarcoding run 4. Species were included from all 11 clades recognised within the *Phytophthora* phylogeny. The DNA concentration (ng/μL) for each species is given in the columns on the right. The darker the colour, the higher the DNA concentration; Figure S2. The number of sequences produced from 10 different primers from mock communities with known DNA concentrations of oomycete species. Each point represents the concentration of an oomycete species and the number of sequences it produced. Each line represents the best-fitted line from the negative binomial generalised linear model for each primer; Figure S3. Phylogenetic representation based on ITS gene region for each *Phytophthora* species detected in the mock community MIX1 and the eDNA sample E1 and E2, as determined in Illumina run 1. The relative proportion of DNA of each species in MIX1 (as a percentage) and the average relative abundance of reads obtained for each species (as a percentage) are colour-coded as per the legend. The asterisk denotes those species known to be present in the eDNA sample E1 and the hashtag represents those present in sample E2; Figure S4. Phylogenetic representation based on ITS gene region of each *Phytophthora* species detected in the mock community MIX1, the eDNA sample E1 and E2, and the eDNA sample E1 spiked with the mock community MIX1, as determined in Illumina run 2. The relative proportion of DNA of each species in MIX1 (as a percentage) and the average relative abundance of reads obtained for each species (as a percentage) are colour-coded as per the legend. The asterisk denotes those species known to be present in the eDNA sample E1 and the hashtag those present in sample E2. Figure S5. Phylogenetic representation based on the ITS gene region of each *Phytophthora* species detected in the mock community MIX2, the eDNA sample E3, and the eDNA sample E3 spiked with the mock community MIX2, as determined in Illumina run 3. The relative proportion of DNA of each species in MIX2 (as a percentage) and the average relative abundance of reads obtained for each species (as a percentage) are colour-coded as per the legend. The asterisk denotes those species known to be present in the eDNA sample E3. Figure S6. Phylogenetic representation based on the ITS gene region of each *Phytophthora* species detected in the mock community MIX3, the eDNA sample E4 and the eDNA sample E4 spiked with the mock community MIX2 as determined in Illumina run 4. The relative proportion of DNA of each species in MIX3 (as a percentage) and the average relative abundance of reads obtained for each species (as a percentage) are colour coded as per the legend. The asterisk denotes those species known to be present in the eDNA sample E4.
