# Supplementary material for: Comparison of Primers for the Detection of Phytophthora (and Other Oomycetes) from Environmental Samples
Source: J Fungi (Basel). 2022 Sep 19;8(9):980. doi: 10.3390/jof8090980 (PMC9502258; doi:10.3390/jof8090980)
Supplement: Supplementary file 1 [file jof-08-00980-s001.zip › jof-1832660-supplementary.pdf]

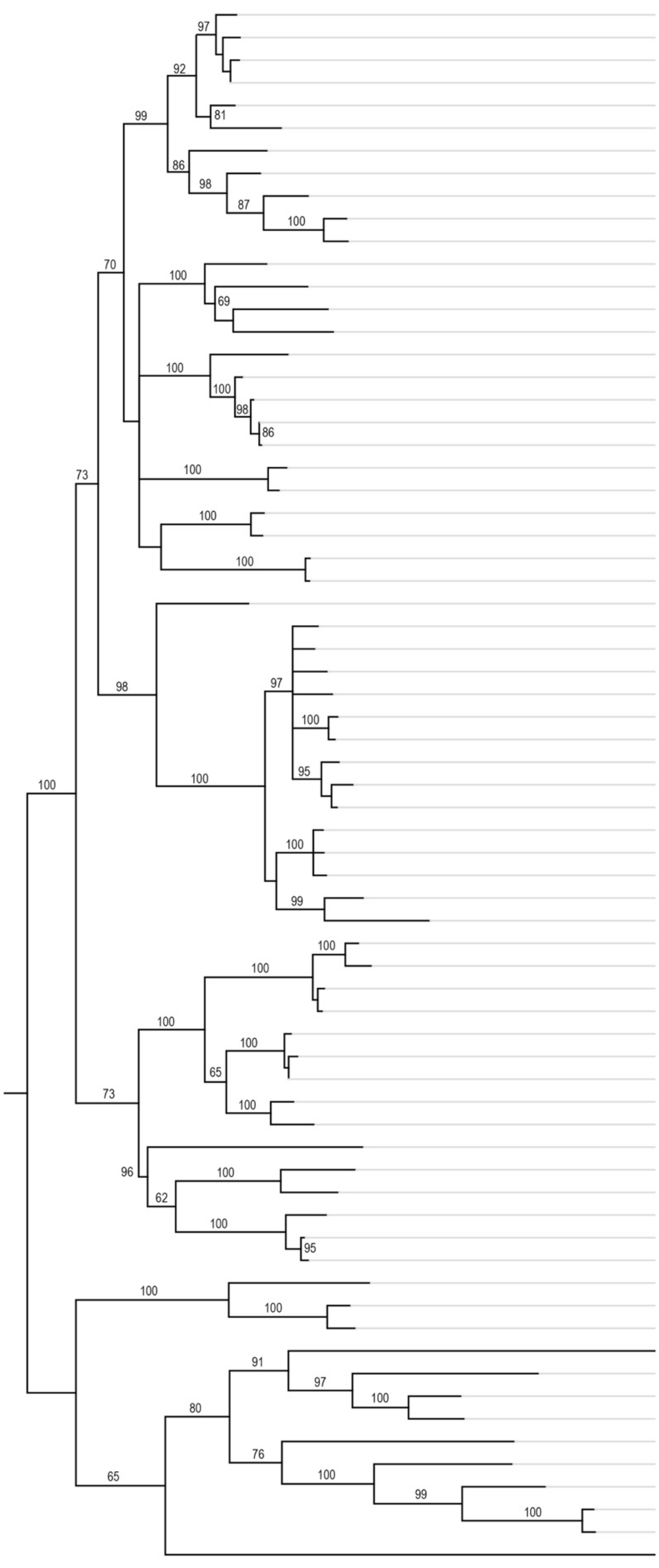

Clade	Species	DNA concentration		
		MIX1	MIX2	MIX3
2	*P. capensis*		2.52	2.60
2	*P. multivora*	66.80	3.21	6.22
2	*P. citricola*	2.90	4.58	0.76
2	*P. plurivora*	12.50	0.76	4.58
2	*P. capsici*		0.29	0.20
2	*P. citrophthora*		0.19	2.90
2	*P. multivesiculata*	2.06	2.02	2.00
2	*P. elongata*	8.40	5.68	6.10
2	*P. bishii*	0.65	0.82	0.85
2	*P. acaciivora*	20.40	2.10	3.10
2	*P. frigida*	3.52	3.58	3.60
1	*P. cactorum*	0.36		
1	*P. infestans*	9.12	6.88	
1	*P. tentaculata*		0.82	0.82
1	*P. nicotianae*	13.70	3.02	3.30
4	*P. palmivora*	15.15	3.66	3.70
4	*P. quercetorum*	14.80	2.46	2.56
4	*P. arenaria*	3.28	4.10	4.00
4	*P. alticola*	0.25	0.20	
4	*P. boodjera*	7.75	1.66	1.70
5	*P. castaneae*	4.42	4.68	3.68
5	*P. heveae*	14.30	2.52	2.52
3	*P. pluvialis*		0.71	0.71
3	*P. psuedosyringae*	3.04	4.06	4.06
12	*P. quercina*	4.78	5.54	6.04
12	*P. versiformis*	9.67	1.91	1.87
6	*P. asparagi*	9.26	9.18	8.80
6	*P.* AUS6H		2.72	0.72
6	*P. bilorbang*	3.76	3.92	3.94
6	*P. ornamentata*		0.29	0.31
6	*P. crassamura*		0.84	0.84
6	*P. gibbosa*	5.04	5.78	2.78
6	*P. gregata*	16.80	4.94	2.94
6	*P. amnicola*	11.20	2.74	2.77
6	*P. mooyotj*	0.40	2.28	2.30
6	*P. thermophila*	2.32	5.57	5.40
6	*P. kwongonina*	0.56	0.62	0.65
6	*P. pseudorosacearum*		1.85	1.35
6	*P. rosacearum*	2.46	2.58	2.62
6	*P. inundata*	3.07	3.54	3.77
6	*P. balyanboodja*		0.19	
7	*P. melonis*		0.36	0.32
7	*P. vignae*		0.17	
7	*P. neiderhauseri*	31.14	2.48	5.48
7	*P. sojae*		0.05	0.05
7	*P. rubi*	9.10	3.62	0.35
7	*P. cambivora*	12.04	2.42	2.42
7	*P. x alni*	0.25	0.20	
7	*P. cinnamomi*	6.98	8.10	2.10
7	*P. parvispora*	3.36	2.92	5.92
8	*P. hibernalis*	2.16	1.49	1.39
8	*P. austrocedrae*	8.08	13.50	3.50
8	*P. syringae*		0.13	0.11
8	*P. dreshsleri*	9.64	7.90	7.90
8	*P. psuedocryptogea*	6.50	6.92	6.80
8	*P. kelmanii*		0.89	0.77
10	*P. kernoviae*	1.34	1.52	1.63
10	*P. boehmeriae*		0.43	0.23
10	*P. gondwanense*	0.97	1.01	1.10
9	*P. insolata*		0.39	0.29
9	*P. aquimorbida*	3.02		
9	*P. hydropathica*	17.10	3.60	3.65
9	*P.* AUS9E	1.73	1.57	2.57
9	*P. macrochlamydospora*		0.30	0.30
9	*P.* AUS9C	0.49		
9	*P. constricta*		2.56	2.55
9	*P. captiosa*	2.68	3.06	1.03
9	*P. fallax*	0.14	0.20	0.20
	Phytopythium litorale	2.52	2.36	2.41

Figure S1. Phylogeny based on the ITS gene region of the *Phytophthora* species was used to create the mock communities. Mock community MIX1 was used for metabarcoding runs 1 and 2, mock community MIX2 was used in metabarcoding run 3, and mock community MIX3 was used in metabarcoding run 4. Species were included from all 11 clades recognised within the *Phytophthora* phylogeny. The DNA concentration (ng/μl) for each species is given in the columns on the right. The darker the colour, the higher the DNA concentration.

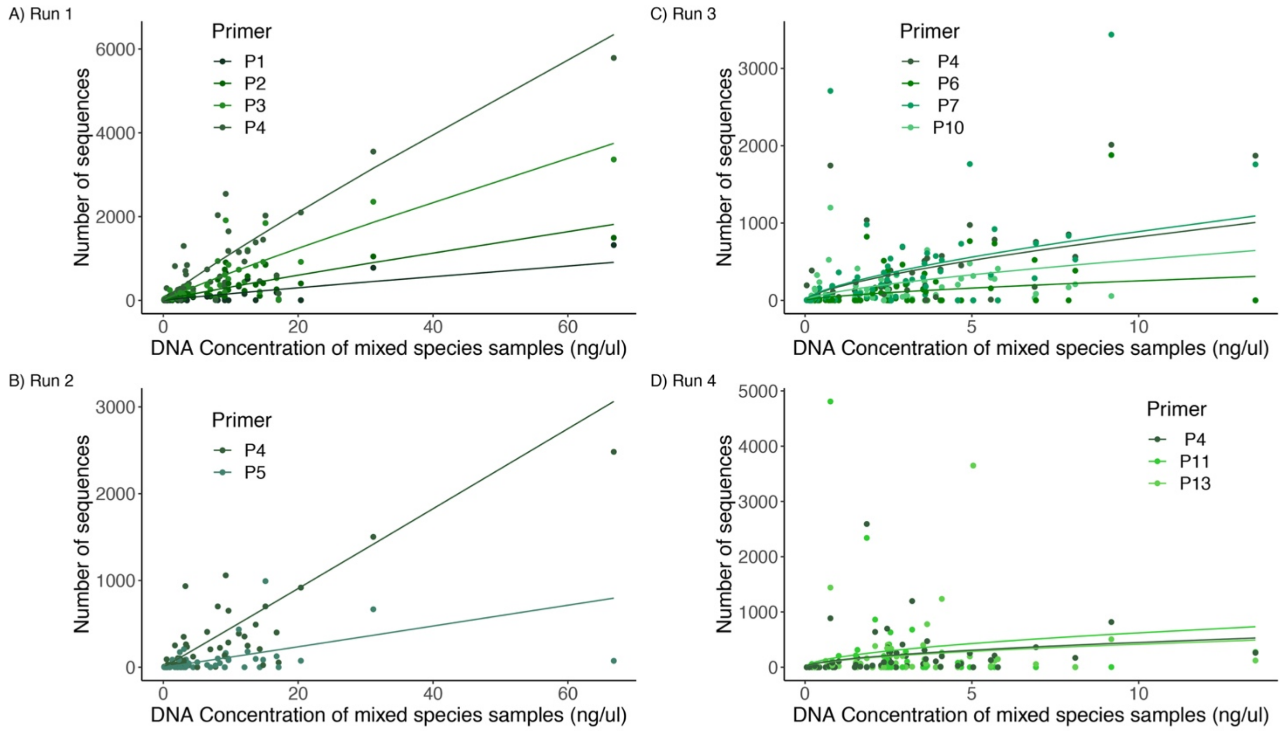

Figure S2. The number of sequences produced from 10 different primers from mock communities with known DNA concentrations of oomycete species. Each point represents the concentration of an oomycete species and the number of sequences it produced. Each line represents the best-fitted line from the negative binomial generalised linear model for each primer.

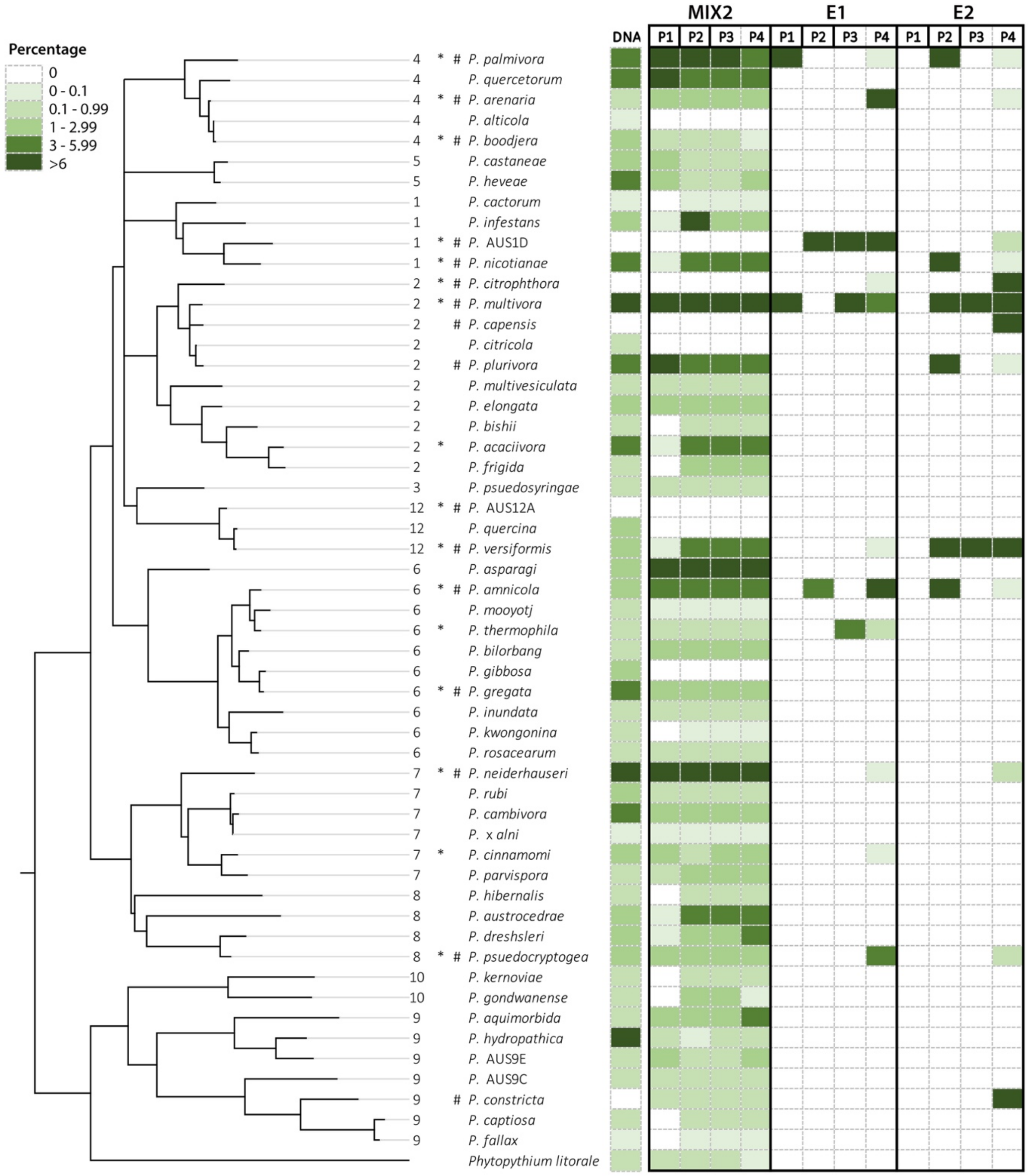

Figure S3. Phylogenetic representation based on ITS gene region for each *Phytophthora* species detected in the mock community MIX1 and the eDNA sample E1 and E2 as determined in Illumina run 1. The relative proportion of DNA of each species in MIX1 (as a percentage) and the average relative abundance of reads obtained for each species (as a percentage) are colour coded as per the legend. The asterisk denotes those species known to be present in the eDNA sample E1 and the hashtag those present in sample E2.

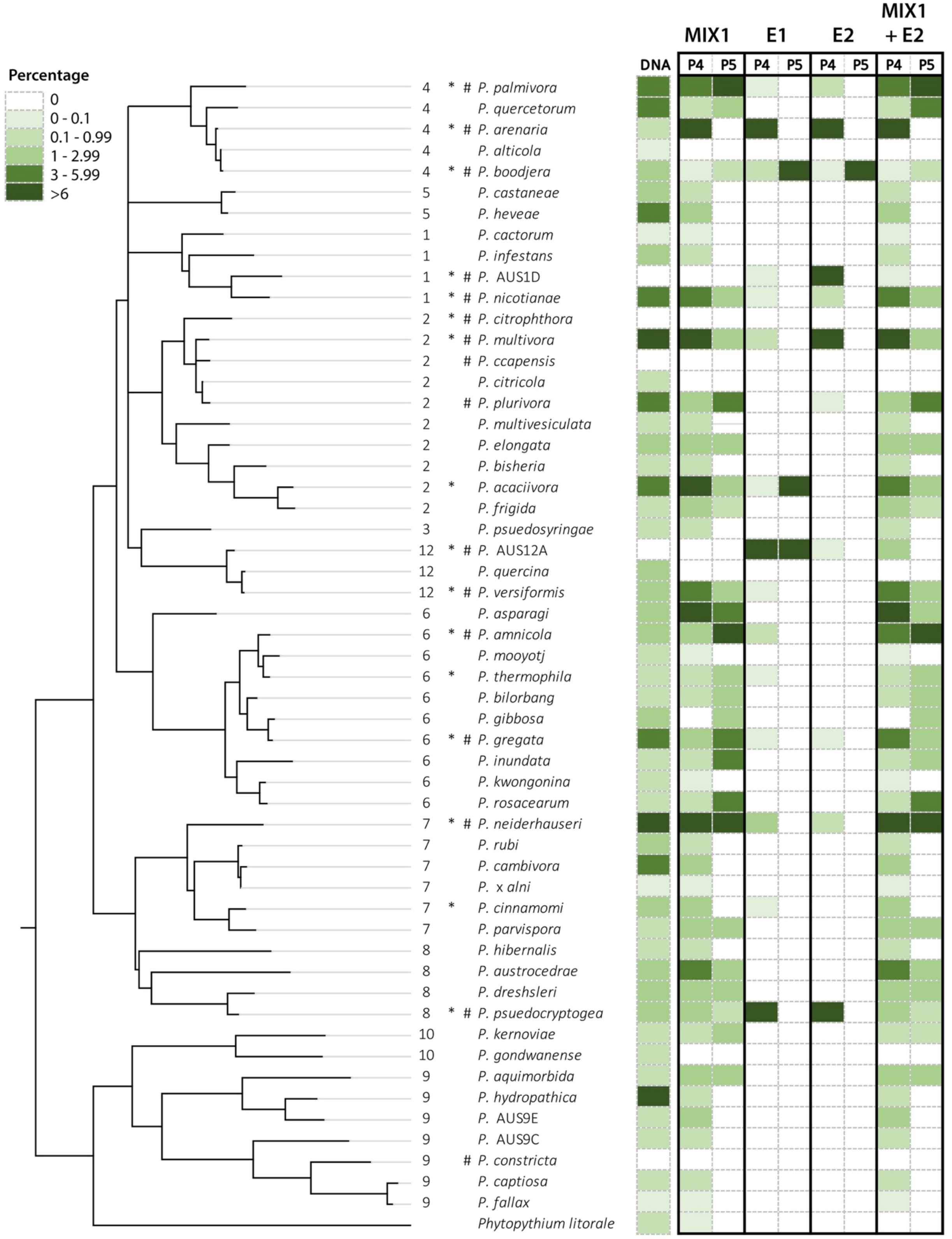

Figure S4. Phylogenetic representation based on ITS gene region of each *Phytophthora* species detected in the mock community MIX1, the eDNA sample E1 and E2 and the eDNA sample E1 spiked with the mock community MIX1 as determined in Illumina run 2. The relative proportion of DNA of each species in MIX1 (as a percentage) and the average relative abundance of reads obtained for each species (as a percentage) are colour coded as per the legend. The asterisk denotes those species known to be present in the eDNA sample E1 and the hashtag those present in sample E2.

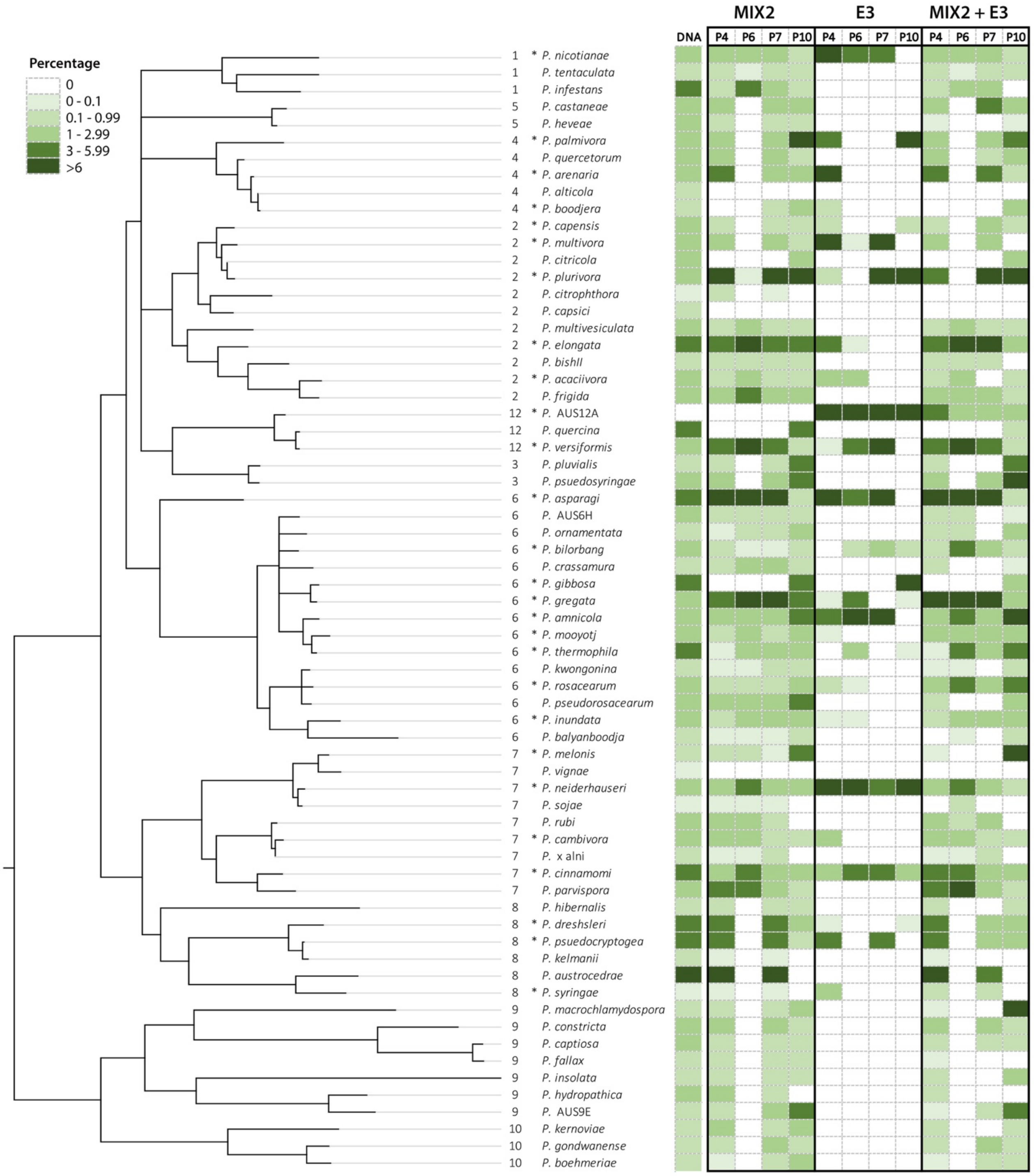

Figure S5. Phylogenetic representation based on the ITS gene region of each *Phytophthora* species detected in the mock community MIX2, the eDNA sample E3 and the eDNA sample E3 spiked with the mock community MIX2 as determined in Illumina run 3. The relative proportion of DNA of each species in MIX2 (as a percentage) and the average relative abundance of reads obtained for each species (as a percentage) are colour coded as per the legend. The asterisk denotes those species known to be present in the eDNA sample E3.

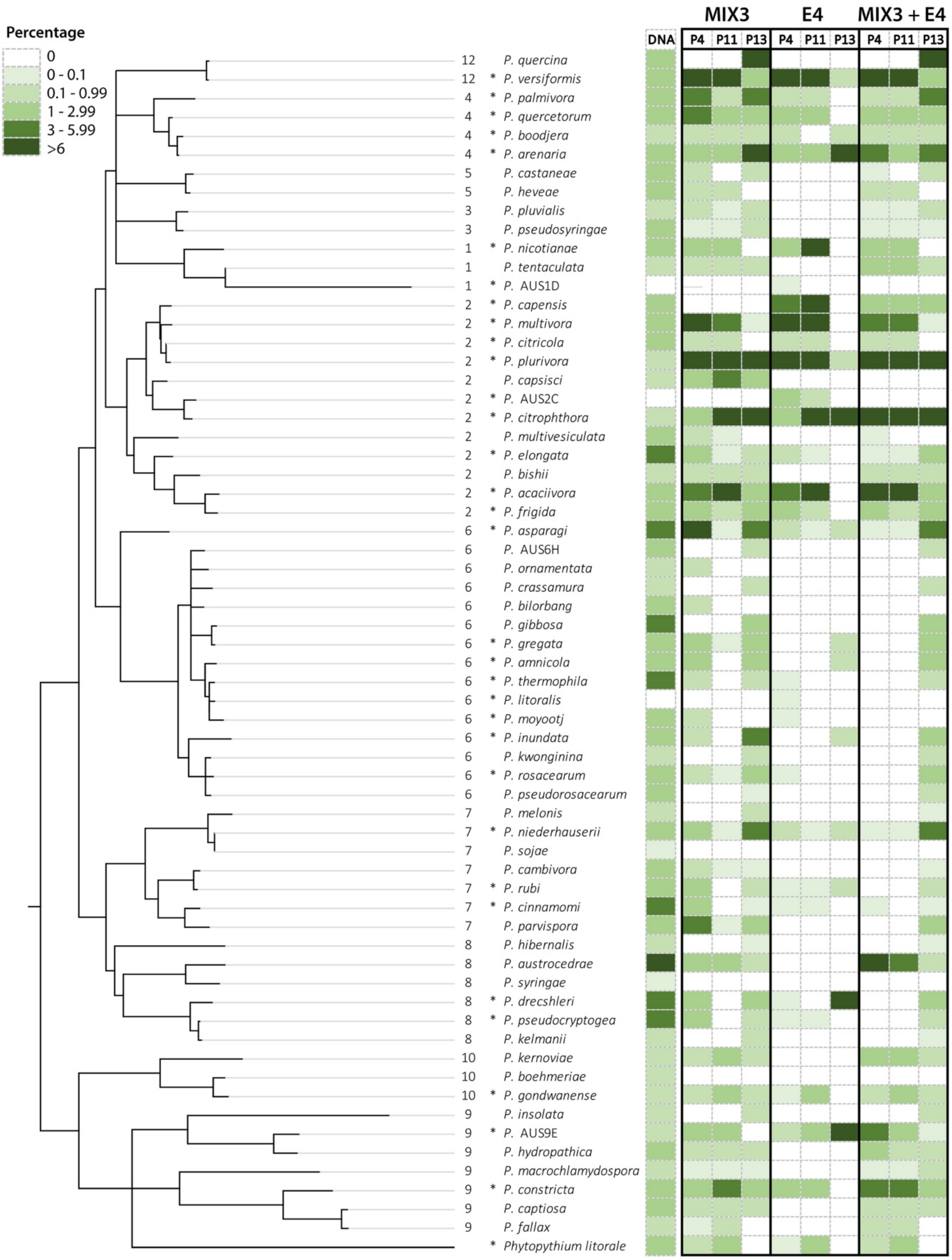

Figure S6. Phylogenetic representation based on the ITS gene region of each *Phytophthora* species detected in the mock community MIX3, the eDNA sample E4 and the eDNA sample E4 spiked with the mock community MIX2 as determined in Illumina run 4. The relative proportion of DNA of each species in MIX3 (as a percentage) and the average relative abundance of reads obtained for each species (as a percentage) are colour coded as per the legend. The asterisk denotes those species known to be present in the eDNA sample E4.
